# Data of a stiffness softening mechanism effect on proliferation and differentiation of a human bone marrow derived mesenchymal stem cell line towards the chondrogenic and osteogenic lineages

**DOI:** 10.1016/j.dib.2018.09.068

**Published:** 2018-09-28

**Authors:** Linxiao Wu, Adrián Magaz, Tao Wang, Chaozong Liu, Arnold Darbyshire, Marilena Loizidou, Mark Emberton, Martin Birchall, Wenhui Song

**Affiliations:** a Centre for Biomaterials in Surgical Reconstruction and Regeneration, Division of Surgery & Interventional Science, University College London, London, United Kingdom; b Precision Medical Centre, the Seventh Affiliated Hospital of Sun Yat-Sen University, Shenzhen 518107, China; c Institute of Orthopaedics and Musculoskeletal Science, Division of Surgery & Interventional Science, University College London, London, United Kingdom; d UCL Ear Institute, Royal National Throat, Nose and Ear Hospital, University College London, London, United Kingdom

## Abstract

This article contains data related to the research article entitled “Stiffness memory of indirectly 3D-printed elastomer nanohybrid regulates chondrogenesis and osteogenesis of human mesenchymal stem cells” [1] (Wu et al., 2018).

Cells respond to the local microenvironment in a context dependent fashion and a continuous challenge is to provide a living construct that can adapt to the viscoelasticity changes of surrounding tissues. Several materials are attractive candidates to be used in tissue engineering, but conventional manufactured scaffolds are primarily static models with well-defined and stable stiffness that lack the dynamic biological nature required to undergo changes in substrate elasticity decisive in several cellular processes key during tissue development and wound healing. A family of poly (urea-urethane) (PUU) elastomeric nanohybrid scaffolds (PUU-POSS) with thermoresponsive mechanical properties that soften by reverse self-assembling at body temperature had been developed through a 3D thermal induced phase transition process (3D-TIPS) at various thermal conditions: cryo-coagulation (CC), cryo-coagulation and heating (CC + H) and room temperature coagulation and heating (RTC + H). The stiffness relaxation and stiffness softening of these scaffolds suggest regulatory effects in proliferation and differentiation of human bone-marrow derived mesenchymal stem cells (hBM-MSCs) towards the chondrogenic and osteogenic lineages.

**Specifications table**

**Table:** 

Subject area	*Chemistry, biology*
More specific subject area	*Biomaterials*
Type of data	*Tables, figures*
How data was acquired	*Static compression and tensile mechanical testing (Instron 5655), Dynamic mechanical compression (ElectroForce Biodynamic® Test Instrument 5160), Mercury intrusion porosimeter (PoreMaster 60GT Quantachrome), Immunohistochemistry, Element detection (EDX, EDAX Inc.)*
Data format	*Analyzed*
Experimental factors	*Compression and tensile mechanical properties in static mode were evaluated with Instron; dynamic compression testing with an ElectroForce bioreactor. The hierarchical porous structure of the scaffolds was analyzed via mercury intrusion porosimeter. Chondrogenic differentiation was studied via Hematoxylin and Eosin, Alcian Blue and Collagen II staining; osteogenic differentiation was studied via Hematoxylin and Eosin, Alizarin Red and Collagen I staining. Energy-dispersive X-ray analysis (EDX) was carried out for elemental mapping analysis.*
Experimental features	*Physico-mechanical characterization, histology and immunohistochemistry*
Data source location	*N/A*
Data accessibility	*Within this article*

**Value of the data**

• Data presented here provides optimized conditions for the assessment of mesenchymal stem cell differentiation on stiffness softening scaffolds.
• Compression mechanical testing along with histological assessment was sensitive to elucidating how stiffness softening affects stem cell differentiation.

## 1 Data

**Fig. 1:**
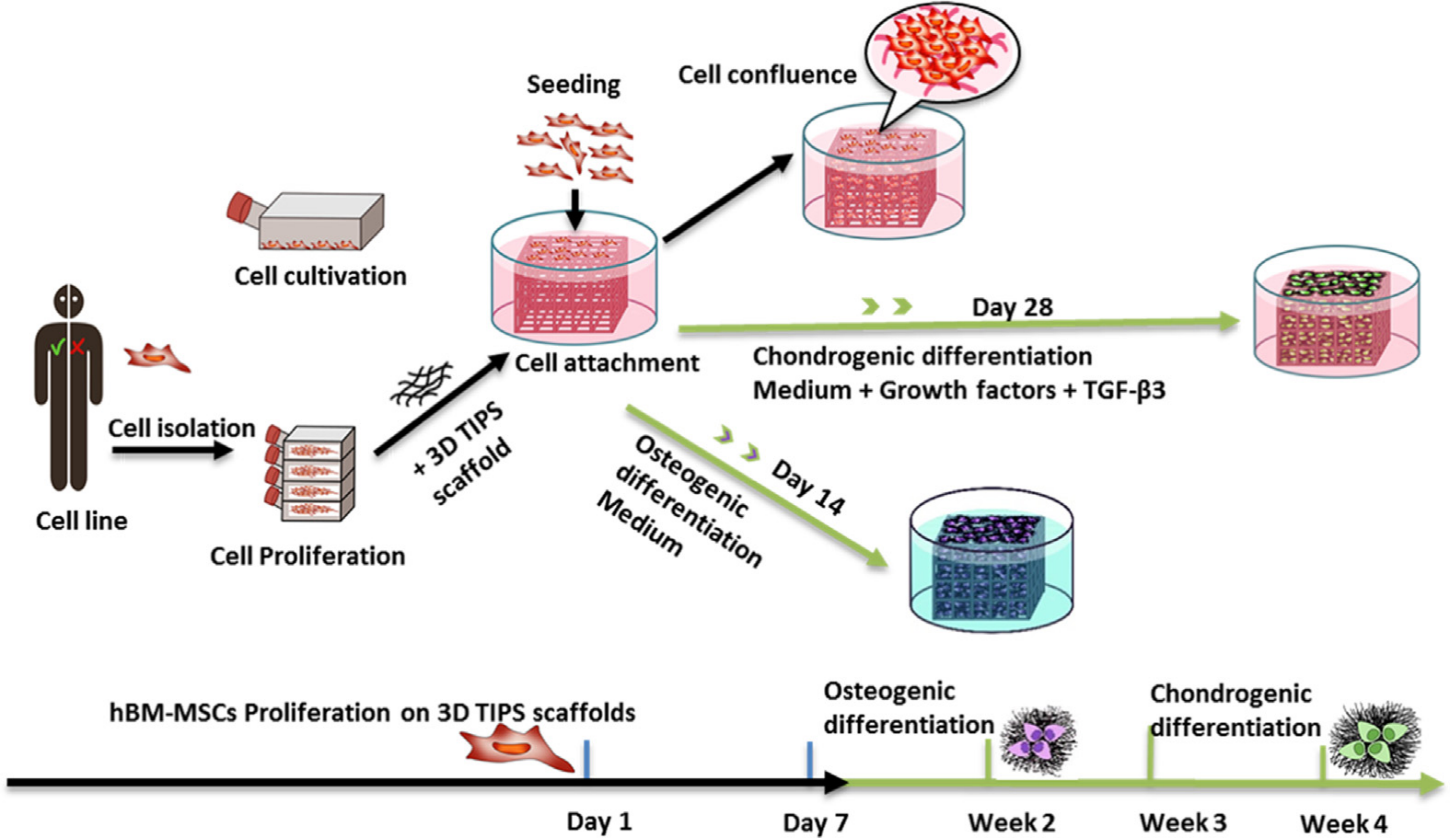
Schematics of hBM-MSCs culture, expansion, seeding and differentiation towards the chondrogenic and osteogenic lineages.

**Fig. 2:**
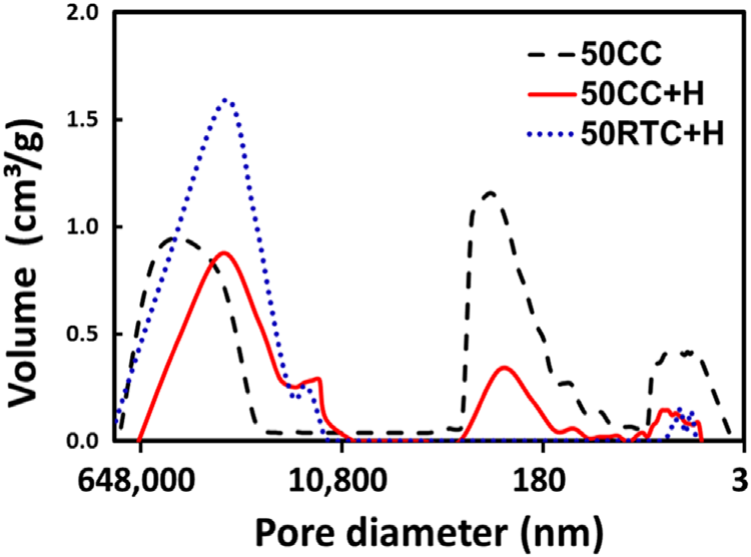
Porosity analysis of 50% infill density scaffolds. Mercury porosimeter measurements in terms of pore size and pore size distribution [2].

**Fig. 3:**
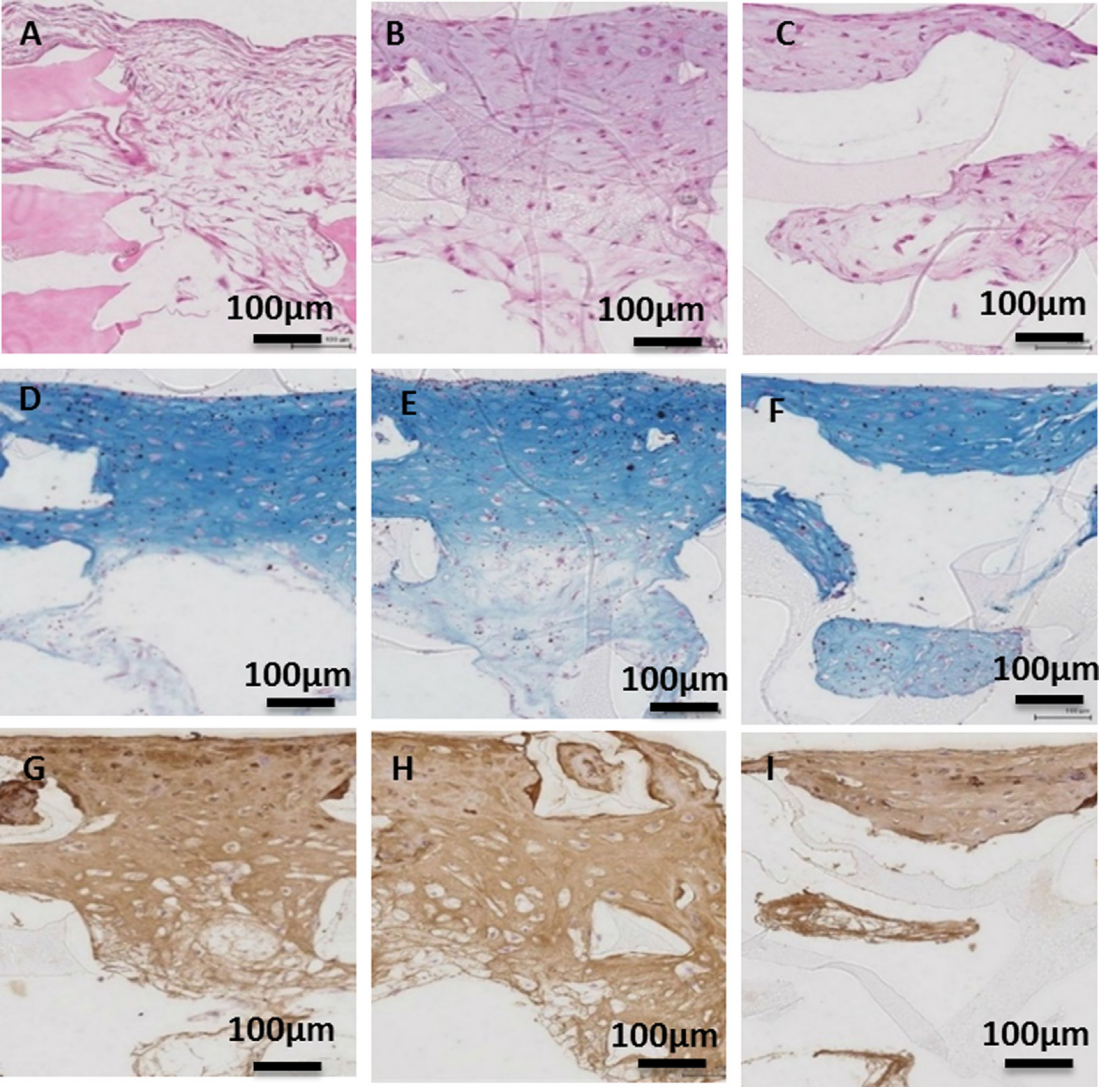
Chondrogenic differentiation on 50% infill density scaffolds. (A-I) Histological analysis of chondrogenic differentiation at week 4: in cross-section for (A, D, G) 50CC, (B, E, H) 50CC+H, and (C, F, I) 50RTC+H. Stained with Hematoxylin and Eosin (H&E), Alcian Blue (A-Blue) and Collagen II (COL2). (For interpretation of the references to color in this figure legend, the reader is referred to the web version of this article).

**Fig. 4:**
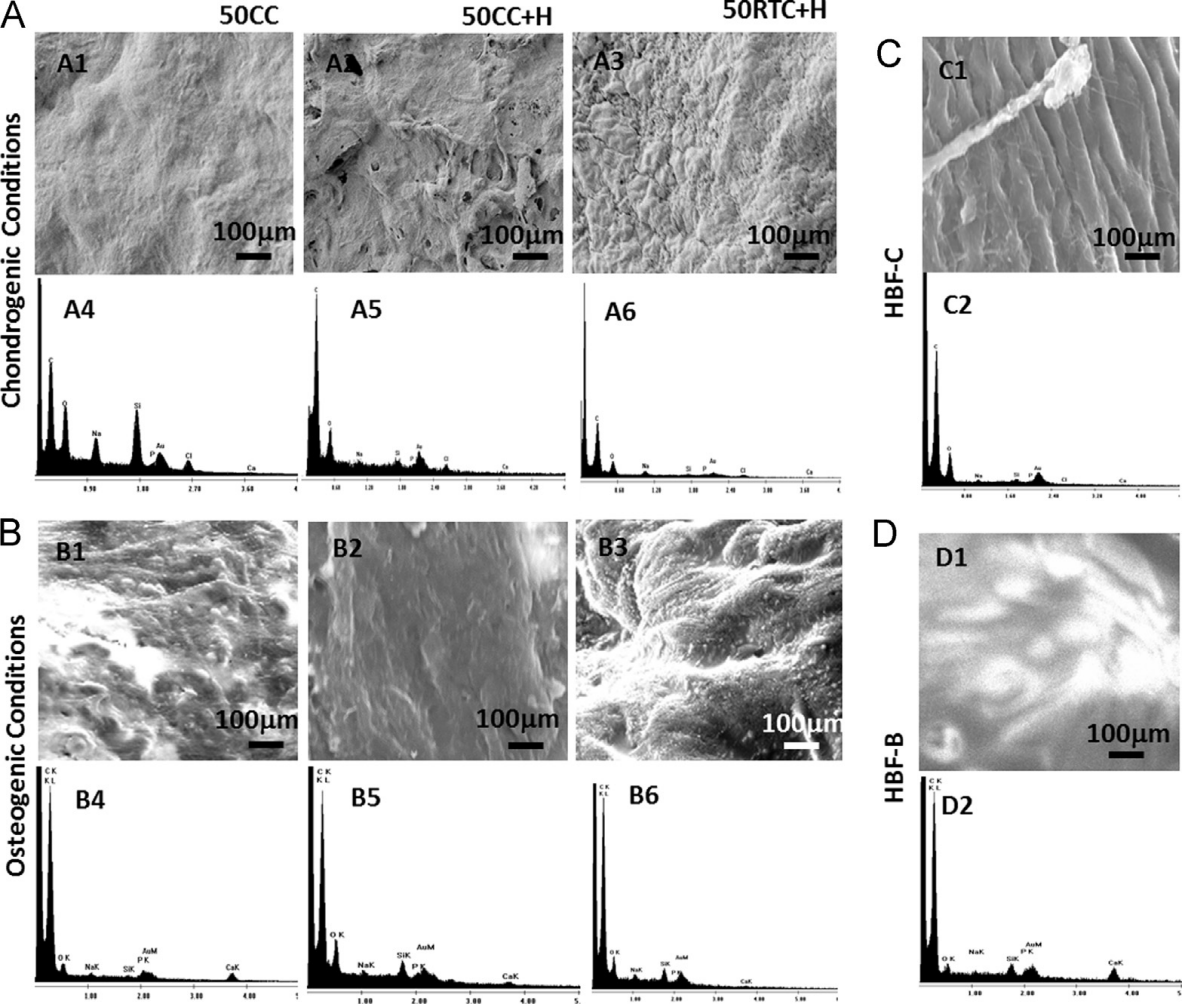
SEM and EDX imaging of hBM-MSCs cultured on the various 50% infill density scaffolds under chondrogenic and osteogenic conditions: (A) after 28 days chondrogenesis on 50CC, 50CC+H and 50RTC+H scaffolds; (B) after 21 days osteogenesis on 50CC, 50CC+H and 50RTC+H scaffolds; (C) human femoral head cartilage control; (D) human femoral head bone control. Scale bar 100 μm.

**Fig. 5:**
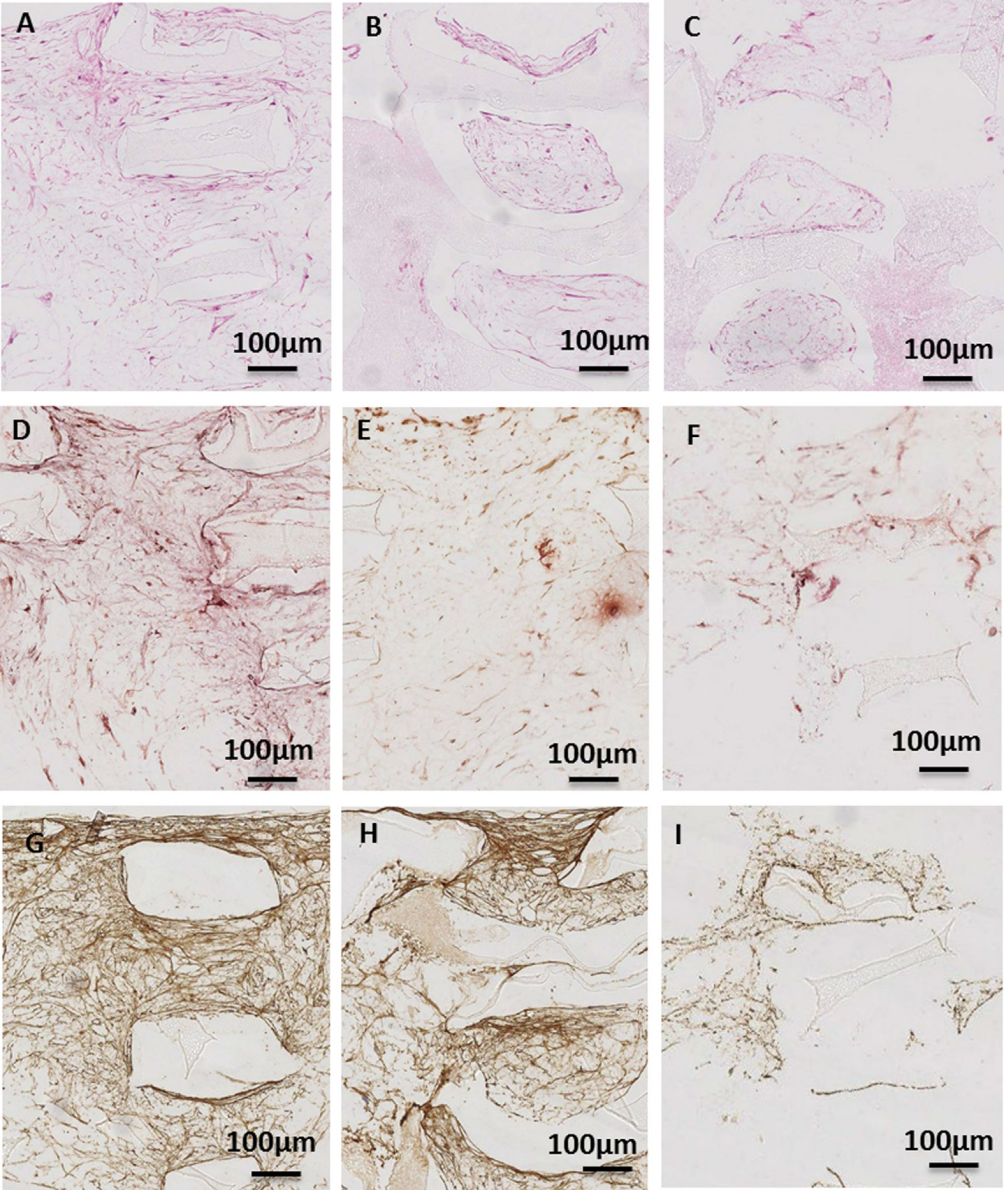
Osteogenic differentiation on 50% infill density scaffolds. (A-I) Histological analysis of osteogenic differentiation at week 4: in cross-section for (A, D, G) 50CC, (B, E, H) 50CC+H, and (C, F, I) 50RTC+H. Stained with H&E, Alizarin red and Collagen I (COL1). (For interpretation of the references to color in this figure legend, the reader is referred to the web version of this article).

Fig. 1 depicts cell expansion and differentiation of human bone-marrow derived mesenchymal stem cells (hBM-MSCs) on 3D-TIPS PUU-POSS scaffolds exhibiting stiffness softening. Table 1 shows the effect of the infill density (i.e. 3D priting) and the variou 3D-TIPS thermal conditions (i.e. CC, CC + H and RTC + H) on the mechanical properties of the scaffolds. Table 2 demonstrates the isothermal stiffness softening behaviour of 50% infill density scaffolds after a 28-day period incubation *in vitro* at body temperature (37°C), with all scaffold groups reaching their intrinsic elasticity (i.e. ׳stiffness memory׳ concept). Table 3 shows viscoelastic behaviours of 50% infill density scaffolds during dynamic compression testing, all reaching their intrinsic elasticity. Fig. 2 and Table 4 demonstrate the hierarchical micro-/nano- porous structure of the various scaffold groups. Figs. 3 and 5 show histological sectioning demonstrating chondrogenic and osteogenic differentiation, respectively, on the various scaffolds. Fig. 4 and Tables 5 and 6 show elemental mapping analysis after chondrogenic and osteogenic differentiation on the various scaffolds.

**Table 1:** Physical, tensile and compression mechanical properties of 3D-TIPS PUU-POSS scaffolds with various infill densities.

**Scaffold**	**Infill density, %**	**Scaffold density, d**_**a**_**, kg/m**^**−3**^	**Total porosity, 100%**	**Compression strength, MPa**	**Compression modulus, MPa**
**CC**	80	44 ± 3	96.2 ± 0.3	0.54 ± 0.02	0.82 ± 0.03
**CC**	70	40 ± 3	96.5 ± 0.3	0.48 ± 0.01	0.75 ± 0.01
**CC**	60	37 ± 5	96.8 ± 0.4	0.34 ± 0.01	0.63 ± 0.02
**CC**	50	36 ± 4	96.9 ± 0.4	0.33 ± 0.03	0.48 ± 0.08
**CC**	40	30 ± 6	97.4 ± 0.5	0.17 ± 0.04	0.39 ± 0.03
**CC**	30	27 ± 3	97.7 ± 0.3	0.10 ± 0.02	0.25 ± 0.02
**CC+H**	80	56 ± 8	95.1 ± 0.7	0.38 ± 0.01	0.56 ± 0.01
**CC+H**	70	51 ± 4	95.5 ± 0.3	0.34 ± 0.04	0.41 ± 0.02
**CC+H**	60	49 ± 3	95.8 ± 0.3	0.26 ± 0.02	0.37 ± 0.03
**CC+H**	50	45 ± 5	96.1 ± 0.5	0.21 ± 0.01	0.27 ± 0.03
**CC+H**	40	41 ± 4	96.5 ± 0.3	0.11 ± 0.01	0.20 ± 0.02
**CC+H**	30	37 ± 2	96.8 ± 0.2	0.13 ± 0.01	0.12 ± 0.01
**RTC+H**	80	48 ± 10	95.8 ± 0.8	0.35 ± 0.01	0.28 ± 0.01
**RTC+H**	70	43 ± 4	96.2 ± 0.4	0.25 ± 0.02	0.26 ± 0.02
**RTC+H**	60	39 ± 5	96.6 ± 0.4	0.22 ± 0.01	0.22 ± 0.01
**RTC+H**	50	38 ± 3	96.7 ± 0.3	0.17 ± 0.02	0.15 ± 0.03
**RTC+H**	40	33 ± 5	97.1 ± 0.4	0.12 ± 0.01	0.13 ± 0.01
**RTC+H**	30	29 ± 3	97.5 ± 0.3	0.10 ± 0.01	0.10 ± 0.01

**Table 2:** Physical and mechanical properties of 3D-TIPS PUU-POSS scaffolds (50% infill density) before and after incubation at body temperature (37°C) for 28 days.

**3D-TIPS scaffold, 50% infill**		**Scaffold density, kg/m**^**3**^	**Total porosity, 100%**	**Young׳s modulus, MPa (Tensile)**	**Ultimate tensile strength, MPa (Tensile)**	**Ultimate tensile strain, % (Tensile)**	**Toughness, J m**^**−3**^** × 10**^**4**^**(Tensile)**	**Compression strength@25%, MPa**	**Compression modulus@25%, MPa**
**50CC**	Day 0	36 ± 4	96.9 ± 0.4	0.98 ± 0.14	1.33 ± 0.09	179 ± 8	137 ± 22	0.33 ± 0.02	0.51 ± 0.08
	Day 28	29 ± 4	97.4 ± 0.3	0.45 ± 0.08	0.77 ± 0.15	230 ± 13	115 ± 20	0.18 ± 0.03	0.16 ± 0.01
**50CC+H**	Day 0	45 ± 5	96.1 ± 0.5	0.53 ± 0.02	0.76 ± 0.05	236 ± 19	113 ± 27	0.22 ± 0.04	0.27 ± 0.03
	Day 28	39 ± 5	96.7 ± 0.4	0.39 ± 0.09	0.72 ± 0.12	240 ± 18	110 ± 14	0.17 ± 0.02	0.13 ± 0.01
**50RTC+H**	Day 0	38 ± 3	96.7 ± 0.3	0.44 ± 0.06	0.67 ± 0.03	146 ± 15	146 ± 12	0.17 ± 0.05	0.15 ± 0.01
	Day 28	32 ± 3	97.2 ± 0.3	0.42 ± 0.08	0.65 ± 0.06	149 ± 19	146 ± 20	0.17 ± 0.02	0.12 ± 0.01

**Table 3:** Hysteresis values (i.e. energy loss) of the various scaffolds (50% infill density) during tensile and compression cyclic loading at day 0 and after incubation for 28 days at 37°C.

**Type of test**	**Day**	**Hysteresis energy (J/m**^**3**^**)**	**50CC**	**50CC+H**	**50RTC+H**
**Tensile**	**D0**	0–200 cycles	160 ± 11	21 ± 8	15 ± 7
		1000–1200 cycles	133 ± 1	18 ± 2	13 ± 2
		10,000–10,200 cycles	24 ± 8	14 ± 3	13 ± 4
		200,000–200,100 cycles	15 ± 5	8 ± 3	11 ± 4
	**D28**	0–200 cycles	31 ± 6	17 ± 4	12 ± 4
		1000–1200 cycles	17 ± 6	13 ± 5	10 ± 2
		10,000–10,200 cycles	12 ± 5	10 ± 4	9 ± 3
		200,000–200,200 cycles	10 ± 4	10 ± 4	9 ± 3
**Compression**	**D0**	0–200 cycles	274 ± 7	125 ± 10	12 ± 4
		1000–1200 cycles	124 ± 9	91 ± 10	14 ± 4
		10,000–10,200 cycles	101 ± 10	81 ± 4	13 ± 4
		100,000–100,200 cycles	90 ± 8	80 ± 4	12 ± 4
		200,000–200,100 cycles	60 ± 5	52 ± 3	10 ± 4
	**D28**	0–200 cycles	63 ± 5	56 ± 8	8 ± 3
		1000–1200 cycles	43 ± 5	35 ± 9	10 ± 5
		10,000–10,200 cycles	31 ± 5	23 ± 7	10 ± 4
		100,000–100,200 cycles	13 ± 5	15 ± 4	8 ± 4
		200,000–200,100 cycles	10 ± 4	9 ± 4	8 ± 3

**Table 4:** Pore size and pore size distribution of 50% infill density scaffolds [2].

**Scaffold**	**Pore diameter, nm**	**Pore volume, cm**^**3**^**/g**	**Relative pore volume, %**	**Surface area, m**^**2**^**/g**	**Relative surface area, %**
**50CC**	456,882–1000	29.75	58.46	1.55	2.65
	1000–100	11.25	22.10	48.67	83.16
	100–3	9.89	19.44	8.30	14.19
**Total**		50.89	100	58.52	100
**50CC+H**	439,998–1000	31.31	75.75	1.47	6
	1000–100	6.57	15.89	18.82	76.84
	100–3	3.45	8.36	4.2	17.16
**Total**		41.33	100	24.49	100
**50RTC+H**	387,810–1000	48.64	95.16	2.68	58.51
	1000–100	0	0	0	0
	100–3	2.47	4.84	1.9	41.49
**Total**		51.11	100	4.58	100

**Table 5:** EDX element analysis of scaffolds (50% infill density) after day 28 chondrogenesis. (weight %, wt%; atomic concentration%, at%)

**Element**	**HFH-C**	**HFH-C**	**50CC**		**50CC+H**		**50RTC+H**	
	**wt%**	**at%**	**wt%**	**at%**	**wt%**	**at%**	**wt%**	**at%**
**C**	65.64	79.76	69.5	83.55	68.73	80.01	70.51	83.55
**O**	17.60	16.01	18.42	14.7	20.42	17.92	16.42	14.70
**Na**	2.37	1.50	0.66	0.41	1.74	1.06	0.66	0.41
**Si**	1.37	1.19	0.61	0.36	0.37	0.19	0.71	0.36
**P**	0.67	0.31	0.36	0.12	0.67	0.18	0.26	0.12
**Ca**	0.92	0.38	0.28	0.03	0.92	0.05	0.08	0.03
**Au**	11.43	0.84	8.36	0.83	11.43	0.58	11.35	0.83
**Total**	100%	100%	100%	100%	100%	100%	100%	100%

HFH-C, human femoral head cartilage.

**Table 6:** EDX element analysis of the scaffolds (50% infill density) after 21 days osteogenesis. (weight, wt%; atomic concentration%, at%)

**Element**	**HFH-B**	**HFH-B**	**50CC**		**50CC+H**		**50RTC+H**	
	**wt%**	**at%**	**wt%**	**at%**	**wt%**	**at%**	**wt%**	**at%**
**C**	77.62	88.99	83.63	91.44	61.27	73.34	69.23	84.03
**O**	8.21	7.06	7.11	5.86	22.67	21.12	15.55	14.17
**Na**	0.71	0.42	0.65	0.37	3.49	2.27	0.63	0.40
**Si**	1.43	0.70	0.24	0.11	2.38	1.86	0.33	0.17
**P**	0.83	0.37	1.27	0.54	0.36	0.17	0.22	0.10
**Ca**	3.72	1.93	2.66	0.88	1.48	0.61	0.29	0.11
**Au**	7.48	0.52	4.45	0.30	8.35	0.63	13.74	1.02
**Total**	100%	100%	100%	100%	100%	100%	100%	100%

HFH-B, human femoral head bone.

### 1.1 Cell expansion and differentiation of hBM-MSCs on 3D-TIPS PUU-POSS scaffolds

See Fig. 1.

### 1.2 Physico-mechanical characterization and ׳stiffness memory׳ of 3D-TIPS PUU-POSS scaffolds

See Table 1, Table 2, Table 3, Table 4 and Fig. 2.

### 1.3 Chondrogenic and osteogenic evaluation

See Fig. 3, Fig. 4, Fig. 5 and Tables 5 and 6.

## 2 Experimental design, materials and methods

### 2.1 3D-TIPS PUU-POSS scaffold manufacturing

3D-TIPS PUU-POSS scaffolds at different thermal conditions (Cyo-coagulation, CC; cryo-coagulation and heating, CC + H; and room temperature coagulation and heating, RTC + H) were manufactured by a 3D confined thermal induced phase separation process (3D-TIPS) based on self-assembly, phase transition and phase separation of the polymeric solution at controlled temperatures as described in [1], [2].

### 2.2 Cell expansion and differentiation

A human bone marrow derived mesenchymal stem cell line was expanded, seeded and differentiated (Table S1-S2) on 3D-TIPS PUU-POSS scaffolds with stiffness softening as described in [1].

### 2.3 Physico-mechanical characterization of the scaffolds prior to cell seeding

Static mechanical testing of the scaffolds under tensile and compression mode, for different infill densities, before and after incubation over 28 days at body temperature *in vitr*o (37°C), was performed with an Instron 5655 tester as described previously [1].

A mercury intrusion porosimeter (PoreMaster 60GT, Quantachrome, UK) was used to characterise the pore structure including the pore size, pore volume, size distribution and surface area of freeze-dried scaffolds (50% infill density).

### 2.4 Chondrogenic and osteogenic assessment

Element detection on cell-laden 50% infill density scaffolds after differentiation was quantified via Energy-dispersive X-ray (EDX) analysis as described in [1].

Histological section and staining of the scaffolds (50% infill density) was performed after chondrogenic and osteogenic differentiation as previously described [1].

## Footnotes

Appendix A Supplementary data associated with this article can be found in the online version at https://doi.org/10.1016/j.dib.2018.09.068.
