# Supplementary material for: Data of a stiffness softening mechanism effect on proliferation and differentiation of a human bone marrow derived mesenchymal stem cell line towards the chondrogenic and osteogenic lineages
Source: Data Brief. 2018 Sep 28;21:133–42. doi: 10.1016/j.dib.2018.09.068 (PMC6186969; doi:10.1016/j.dib.2018.09.068)
Supplement: Supplementary file 1 — Supplementary material [file mmc1.docx]

**Competing interests**
The authors declare no potential conflict of interests with respect to the research, authorship and/or publication of this article.
