# Supplementary material for: Data of a stiffness softening mechanism effect on proliferation and differentiation of a human bone marrow derived mesenchymal stem cell line towards the chondrogenic and osteogenic lineages
Source: Data Brief. 2018 Sep 28;21:133–42. doi: 10.1016/j.dib.2018.09.068 (PMC6186969; doi:10.1016/j.dib.2018.09.068)
Supplement: Supplementary file 2 — Supplementary material [file mmc2.docx]

**Supplementary information**

**Data of a stiffness softening mechanism effect on proliferation and differentiation of a human bone marrow derived mesenchymal stem cell line towards the chondrogenic and osteogenic lineages**

Linxiao Wu^1^, Adrián Magaz^1†^, Tao Wang^1,2^, Chaozong Liu^3^, Arnold Darbyshire^1^, Marilena Loizidou^1^, Mark Emberton^1^, Martin Birchall^4^, Wenhui Song^1*^

^1^Centre for Biomaterials in Surgical Reconstruction and Regeneration, Division of Surgery & Interventional Science, University College London, London, United Kingdom

^2^Precision Medical Centre, the Seventh Affiliated Hospital of Sun Yat-Sen University, Shenzhen 518107, China

^3^Institute of Orthopaedics and Musculoskeletal Science, Division of Surgery & Interventional Science, University College London, London, United Kingdom

^4^UCL Ear Institute, Royal National Throat, Nose and Ear Hospital, University College London, London, United Kingdom

* Corresponding author, email: [w.song@ucl.ac.uk](mailto:w.song@ucl.ac.uk)

† Current address: Bio-Active Materials Group, School of Materials, The University of Manchester, Manchester, UK

**Table S1** List of primers used for qPCR (chondrogenesis)

| \| **Gene** \| \| --- \| | **Primer sequence (sense/antisense)** | **Tm (°C)** |
| --- | --- | --- | --- |
| *SOX9* | 5’-GCCTTTTTGTCCATCCCTTTTTTC-3’  5’-GTCCTTGGGGTTCTTGCTGATGTA-3’ | 64.6  65.3 |
| *COL2A1* | 5’-ACCTCACGCCTCCCCATCATTG-3’  5’-ACATCAGGTCAGGTCAGCCATTCAG-3’ | 62.0  62.6 |
| *COL X* | 5’-TGAAAGGGACTCATGTTTGGGTAGG-3’  5’-ACTCACATTGGAGCCACTAGGAATC-3’ | 60.5  60.4 |
| *ACCAN* | 5’-TGAGGAGGGCTGGAACAAGTACC-3’  5’-GGAGGTGCTAATTGCAGGGAACA-3’ | 61.0  62.3 |
| *GAPDH* | 5’-TGATGACATCAAGAAGGTGGTGAAG-3’  5’-TCCTTGGAGGCCATGTGGGCCAT-3’ | 60.0  60.0 |

*SOX9*, transcription factor SOX9; *COL2A1*, collagen type II; *COLX*, collagen type X; *ACAN*, Aggrecan; *GAPDH,* glyceraldehyde phosphate dehydrogenase.

**Table S2** List of primers used for qPCR (osteogenesis)

| \| **Gene** \| \| --- \| | **Primer sequence (sense/antisense)** | **Tm (°C)** |
| --- | --- | --- | --- |
| *ALP* | 5’-GCCTTTTTGTCCATCCCTTTTTTC-3’  5’-GTCCTTGGGGTTCTTGCTGATGTA-3’ | 64.6  65.3 |
| *COL1A1* | 5’-CGCTACTACCGGGCTGATGAT-3’  5’-GTCCTTGGGGTTCTTGCTGATGTA-3’ | 62.0  62.6 |
| *RUNX2* | 5’-AGAGGTACCAGATGGGACTGTGGTT-3’  5’-GGTAGCTACTTGGGGAGGATTTGTG-3’ | 61.76  62.63 |
| *SPP1* | 5’-ACTTGGAAGGGTCTGTGGGGCT-3’  5’-AGGCATCACCTGTGCCATACCA-3’ | 60.5  60.4 |
| *BGLAP* | 5’-ATGAGAGCCCTCACACTCCTC-3’  5’-GCCGTAGAAGCCGATAGGC-3’ | 61.0  62.3 |
| *SP7* | 5’-TGCACTCTCCCTGCCAGACCTC-3’  5’-AACGGGTCCCAAGGAGCCAGG-3’ | 60.0  60.0 |
| *GAPDH* | 5’-TGATGACATCAAGAAGGTGGTGAAG-3’  5’-TCCTTGGAGGCCATGTGGGCCAT-3’ | 60.0  60.0 |

*ALP*, alkaline phosphatase; *COL1A1*, collagen type I; *RUNX2*, cbfa-1; *SPP1*, Osteopontin; *BGLAP*, Osteocalcin; *SP7,* Osteorix; *GAPDH*, glyceraldehyde phosphate dehydrogenase.
